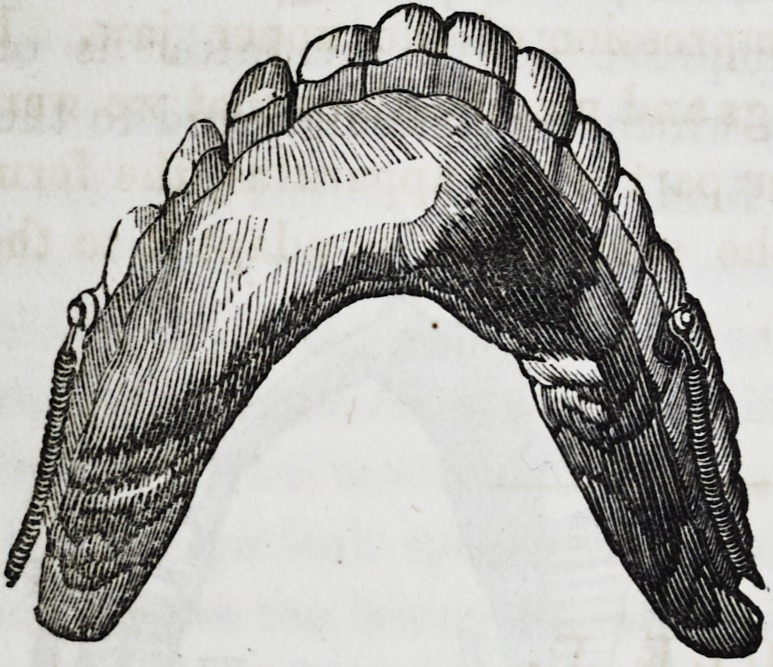# Artificial Piece Made to Replace the Inferior Maxillary Bone
*This piece was presented to the Society of Surgeons, Paris, by MM. Fowler and Preterre, during its meeting on the 3d of Dec., 1856.


**Published:** 1857-04

**Authors:** 


### ARTICLE IV.

*Artificial Piece made to replace the Inferior Maxillary Bone.**
From the *Gazette des Hopitaux* for Dec. 13th, 1856.
Translated for the Am. Jour. of Dent. Science.

THE man whom we have the honor of presenting to the Society, named Jerome Isamat, aged thirty-three, underwent the complete excision of the lower jaw, which was rendered necessary by a large fibrous tumor developed in the interior of this bone, on the 15th of last April at the hospital *de la Pitie.* This patient has been before presented to the Society by M. Maisenneuve, who, in speaking of the fine results of his operation, has also called attention to the artificial piece designed to replace the excised maxillary. This piece was only temporary, as absorption modified each succeeding day, the state of the parts. To-day, the time elapsed since the operation, (seven months,) permits the hope that their form will not further change to any considerable extent, and moreover, the patient having determined to return to his own home, (Barcelona,) we

* This piece was presented to the Society of Surgeons, Paris, by MM. Fowler and Preterre, during its meeting on the 3d of Dec., 1856.

have made for him a permanent piece. This apparatus I now submit to the examination of the members of this Society:—For the details of the operation, we refer you to the observations of M. Maisenneuve, communicated to the Academy of Sciences and Medicine on the 12th and 13th of May, and published in the 39th No. of the *Gazette des Hopitaux;* we would now only make the following remarks. When the patient is deprived of his apparatus, all the lower part of the face is soft, the depressions in the cheek extend to the places occupied by the branches of the inferior maxillary; the chin tapers a little backward, and the lower lip is upon a plane, sensibly posterior to that of the upper. These deformities are, however, pretty well hidden by the beard. Looking into the mouth, we observe a mucous surface, curved and inclined from above downward and from before backward, in such a manner as to form a sort of gutter, limited in front by the border of the lower lip, behind by the base of the tongue, and continuing along the internal parts of the cheek. This gutter is perfectly smooth, presenting no other inequality than a slight ridge elevating the mucous membrane, and having on the right the consistence of a fibrous cord. The ridge is caused by the periosteum, which has been preserved, and by the linear cicatrix of the mucous membrane. The tongue executes all its movements; that of propulsion is, however, a little limited. The movements of the lip are sufficiently easy, but when the patient speaks, the contraction of the muscles of the face, draws the lower lip, which is not supported, considerably backward. The mouth can be readily opened and shut, the opening only being less than in the normal condition; in order to increase this, the patient instinctively applies his tongue against the mucous surface of the lower lip. The pronunciation is quite correct, although Isamat experiences some difficulty in forming the dental consonants; this is particularly evident when he wishes to raise his voice. Mastication is completely impossible. The patient is only able, with the aid of his tongue, to press his food against the upper teeth, thus imperfectly cutting his food.

Wearing his apparatus, Isamat presents a very different aspect. The lower part of his face becomes the *point d'appui;* the lower lip is sustained and the visage recovers its regularity, we observe indeed, two trifling depressions, on each side, over the branches of the maxillary; these could be obviated by new adaptations, but the piece would then be too heavy and inconvenient, and the beard hides them in a measure. Thanks to the support the muscles derive from the elasticity of the apparatus, Isamat does not now find it necesaary to apply his tongue to his lower lip in order to open the mouth. The strength of the springs together with that of its muscles, is sufficient to hold the closed mouth, in a state of repose, and that without causing the patient the slightest effort. With his apparatus he speaks with much greater facility, and not only pronounces the dentals very distinctly but is also able to elevate his voice to any pitch he may desire. Instead of his former imperfect mastication he can now chew substances heretofore impossible, as crusts of bread, meat, fruit, etc., with perfect ease. His pronunciation and digestion improve daily; the latter is of course owing to the facility with which he masticates, and to the more perfect insalivation of his food.

*Description of the piece.* The apparatus is composed of two parts, an upper and a lower, united together by hinges and springs. The upper part (A) is made of a gold plate, 18 millimètres (.70866 of an Eng. in.) in depth, and which fits very accurately the base of the superior dental arch and the anterior part of the roof of the mouth. This plate extends from the second small molar* on the

* According to our classification, the second *bicuspid*.

right side, to the corresponding tooth on the left. The anterior border presents an irregular festooned appearance, caused by the adaptation of the plate to the necks of the remaining teeth. Laterally, the piece is supported by means of clasps, 3 millimètres (.11811 of an Eng. in.) in width, attached to the crowns of the first bicuspids. (G.) The large molars are covered by extra thick plate, fitting superiorly to the molars, inferiorily presenting the triturating surface delineated in the cut (H); it will be observed that there is a corresponding arrangement in the artificial lower jaw. The metallic covering of the molars was found necessary, in order to give the proper degree of solidity to the articulation of the upper and lower parts of the piece; and, moreover, we thought that ordinary artificial teeth applied over the natural ones, would fail to prove as serviceable during the process of mastication, as the *metallic* teeth. We had endeavored to bring together the grinding surfaces of the natural and artificial teeth, but with this patient, the lateral movement being deficient, mastication would have been made imperfect.

The right side does not differ from the left except in the particular, that the first large molar, having been lost from the right side, we have profited by the interval so caused, to place there the spring-holder, (F) which fixes the superior extremity of the spring of this side. To the left, the superior extremity of the spring is fixed on a level with the space existing between the second bicuspids and the first large molar.

*Lower Part.* This part is composed essentially of the *artificial maxillary*, upon which are fixed the mineral gum teeth; each gum tooth forming a separate piece, thus greatly facilitating repair, if any should become necessary. The base is of gold, and of considerable bulk, but being hollow, it is believed that the proper proportion in regard to weight, has been preserved. The whole apparatus does not weigh over 86 grammes. (2oz. 15dwt. 7.324grs. Troy.)

The enclosed cavity is of course hermetically sealed, in order the more effectually to exclude the deposition of food or saliva.

The inferior border of this piece, which represents to a certain extent, the base of the excised bone, is thick and rounded, and which finds a bed, so to speak, in the *gutter* formed by the mucous wall of the lower lip and the floor of the mouth.

One of the greatest difficulties in the above case, was to take the impression; the parts were so soft and yielding that they offered but little resistance, and eventually we found it necessary to rely solely upon the impression of the upper jaw. It was only by a series of gropings and modifications that we were at last able to give to the lower part of the apparatus, the form of the natural jaw, and at the same time to adapt it to the conformation of the soft parts.

## Figures and Tables

**Figure f1:**
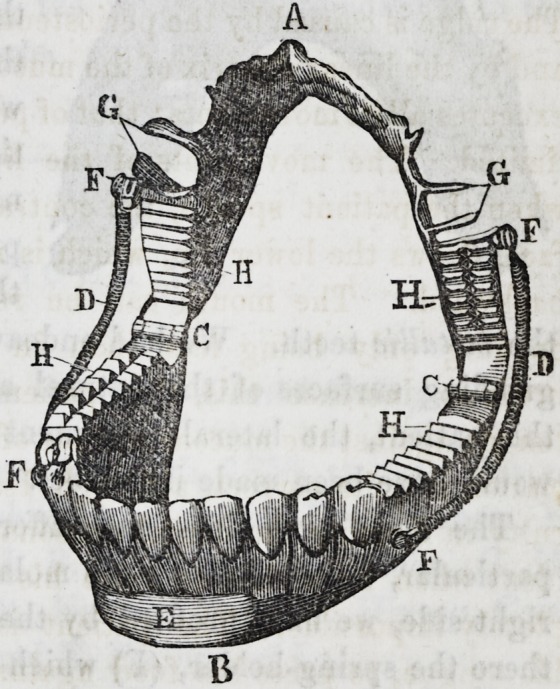


**Figure f2:**
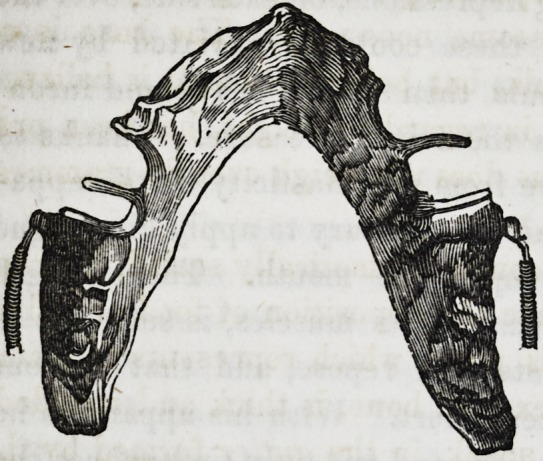


**Figure f3:**